# Spatial and Temporal Distribution of Non-Biting Midge Larvae Assemblages in Streams in a Mountainous Region in Southern Brazil

**DOI:** 10.1673/031.013.15601

**Published:** 2013-12-20

**Authors:** Elzira Cecília Serafini Floss, Elisangela Secretti, Carla Bender Kotzian, Marcia Regina Spies, Mateus Marques Pires

**Affiliations:** 1 Programa de Pós-Graduação em Biodiversidade Animal, Centro de Ciências Naturais e Exatas, Universidade Federal de Santa Maria. Faixa de Camobi km 9, 97105-900, Santa Maria, RS, Brazil; 2 Departamento de Biologia e PPG Biodiversidade Animal, Centro de Ciências Naturais e Exatas, Universidade Federal de Santa Maria. Faixa de Camobi km 9, CEP 97105-900, Santa Maria, RS, Brazil; 3 Universidade Federal do Pampa (Unipampa - Campus São Gabriel) Av. Antônio Trilha 1847, CEP 97300-000, São Gabriel, RS, Brazil; 4 Programa de Pós-Graduação em Biologia, Universidade do Vale do Rio dos Sinos (Unisinos) Av. Unisinos 950, CEP 93022-000, São Leopoldo, RS, Brazil

**Keywords:** aquatic insects, communities, ecology, diversity, Neotropical region

## Abstract

The spatial and temporal structure of non-biting midge (Diptera: Chironomidae) larvae assemblages and some environmental factors that affect their distribution were analyzed in a montane river and its tributaries in a temperate climate region of southernmost Brazil. In total, 69 taxa were recorded after four seasonal samplings (winter, spring, summer, and autumn). The dominant taxa were *Rheotanytarsus* sp. 1, *Rheotanytarsus* sp. 2, *Cricotopus* sp. 2, and *Polypedilum (Polypedilum*) sp., although dominance varied among the four sampling sites. The variations in dominance, abundance, and richness among the different sites were affected by environmental characteristics, such as the presence of marginal vegetation and a heterogeneous substratum, and also by human activities. Strictly environmental factors, such as altitude, and factors related to annual weather patterns, such as mean temperature and precipitation, influenced the spatial and temporal distribution of certain taxa and the structure of faunal assemblages. The influence of the riparian vegetation and riverbed heterogeneity on the composition, richness, and abundance of the chironomid larvae assemblages indicates that human activities, such as deforestation and the construction of dams, constitute a serious threat to the conservation of these insects and to the fauna that depends on them for food.

## Introduction

Non-biting midges (Diptera: Chironomidae) are one of the most diverse and numerous groups among aquatic macroinvertebrates. Their larvae can reach great densities and are the dominant insects in many freshwater environments (Coffman and Ferrington 1996; Paggi 2009). They constitute an important element of the food chain as a food source for several species of fish (Goyke and Hershey 1992; Fagundes et al. 2007), birds (Sánchez et al. 2006), and invertebrates. In addition, many chironomid species are extremely sensitive to specific environmental conditions and therefore are good indicators of water quality. Previous studies show that many taxa can be used to indicate trophic conditions of lakes (Saether 1979), organic and inorganic pollution (Lindergaard 1995a; Al-Shami 2010), environmental hydraulic conditions (Rosa et al. 2011), metal contamination due to coal mining (Bisthoven et al. 2005), etc. Pollution can also produce deformities in larval mouthparts (Bisthoven et al. 2005). Many of these factors, as well as urbanization and the accompanying environmental degradation (Carew 2007; Gresens et al. 2007; Koperski 2009; Al-Shami et al. 2010), also cause changes in the structure of the chironomid larvae assemblages. Assemblages of non-biting midge larvae, as well as other aquatic organisms, have been severely affected by human activities that alter the water quality of freshwater ecosystems.

The structure and the spatial and temporal distribution of chironomid larvae assemblages are strongly influenced by several environmental factors. Among these are many local spatial-scale factors, such as oxygen, substrate, hydraulic conditions, aquatic vegetation, pH, temperature, altitude, and nutrient dynamics (e.g., Saether 1979; Cranston 1995; Stevens et al. 1998; Ali et al. 2002; Reynolds and Benke 2005; Woodcock et al. 2005; Principe et al. 2008; Punti et al. 2009; Roque et al. 2010; Luoto 2011). Wider spatial-scale factors (e.g., landscape, regional) can also affect the assemblages (Martel et al. 2007; Bonada et al. 2008), but small-scale factors seem to play a more important role in their distribution (Ali et al. 2002; Bisthoven et al. 2005; Inoue et al. 2005; Woodcock et al. 2005; Rios and Bailey 2006; Principe et al. 2008; Al-Shami et al. 2010; Floss et al. 2012). Studies of the short-term temporal distribution (small-scale, approximately one year) of chironomids are scarce and show that temperature and rainfall are related to fluctuations in the structure of larvae assemblages (e.g., Siqueira et al. 2008; Chaib et al. 2011). Therefore, comprehending how chironomid assemblages are structured and distributed spatially and temporally, according to the environmental factors, constitutes an important step in preserving the diversity of this insect family.

Most studies done in Brazil have focused on the ecology of chironomid larvae assem-blages concentrated in the southeast region of the country, where tropical to subtropical climates predominate (e.g., Marques et al. 1999; Sanseverino and Nessimian 2008; Simião-Ferreira et al. 2009; Roque et al. 2010; Rosa et al. 2011). However, a recent inventory has shown that the richest (approximately 100 taxa) fauna of the country occurs in a watershed located in a mountainous region of southernmost Brazil (Floss et al. 2012), where the climate is considered temperate. The southern Brazilian region has a rich hydrographic network that has been exploited for agricultural activities (Beskow 1984). River damming for various purposes is also common (Müller 1995). A recent regional (state) law (Portaria SEMA/FEPAM, No. 94, 16 December 2008) allows the construction of small reservoirs for agricultural purposes with no need for environmental assessment. In other words, lotic environments are becoming lentic, and therefore the impacts on the riverine fauna and flora must be properly studied. This study analyzed the spatial and temporal structure of the chironomid larvae assemblages in the middle Jacuí River basin, the region previously inventoried by Floss et al. (2012). The influence of environmental factors on the spatial and temporal distribution of the assemblages was also analyzed using a small-scale approach.

## Materials and Methods

### Study area

The Jacuí River basin is one of the most important watersheds in southern Brazil. It covers approximately 71,000 km^2^ of drainage area and is 710 km long (Zamanillo et al. 1989). The basin has been intensely modified for agriculture, livestock, energy production, navigation, and urban water supplies. The middle part of the basin is located on the Lower Northeast Slope, a mountainous region between the Plateau and the lowlands of the Central Depression, with altitudes from 50 to 500 m (Pereira et al. 1989). The valley is deep and straight, and the Jacuí River, as well as its tributaries, has a rocky bed consisting mainly of boulders and pebbles (Neri et al. 2005) with little aquatic vegetation (Spies et al. 2006). Near the lower course, the Jacuí is dammed by the Dona Francisca Hydroelectric Power Station (29° 26′ 50″ S; 53° 16′ 50″ W).

The mean temperature ranges from 18° C to 22° C during the summer and reaches 13° C during the winter, so some specialists consider the regional climate to be temperate (Maluf 2000). Rain is regularly distributed throughout the year, with the mean annual rainfall varying from 1,500 to 1,708 mm (Pereira et al. 1989; Maluf 2000). The seasonal deciduous forest was the original vegetation of the region. Nowadays it is vastly altered and contains small portions of secondary riparian vegetation (Durlo et al. 1982; Longhi et al. 1982; Marchiori et al. 1982).

### Sampling sites

**Figure 1.**
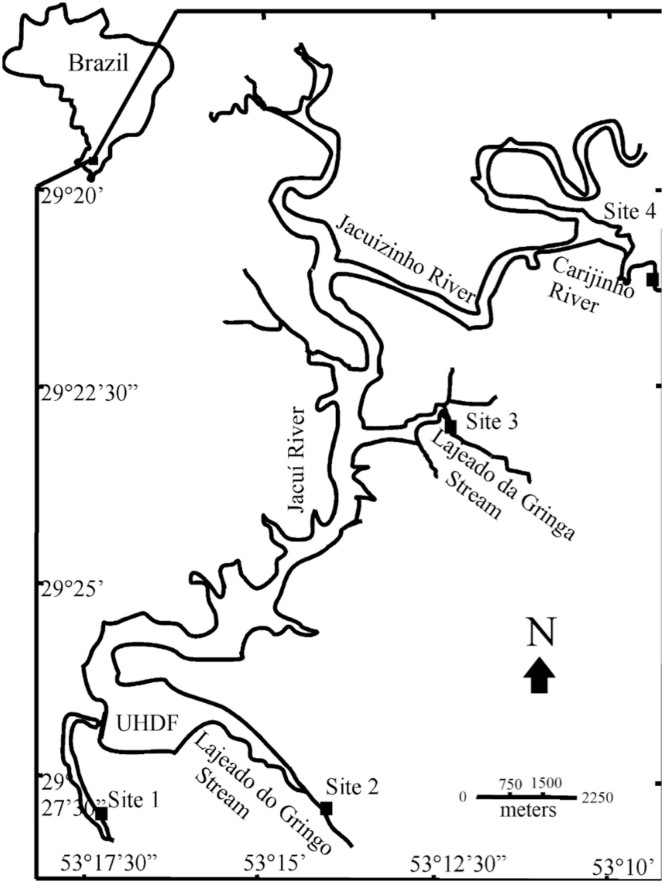
Map of the area of study, indicating the four sampling sites in the middle course of the Jacuí River Basin, in the state of Rio Grande do Sul (RS), Brazil. Adapted from Spies et al. (2006). High quality figures are available online.

Four sampling sites located in an environmentally protected area (Parque Estadual da Quarta Colônia), which was created to compensate for the impacts caused by the hydroelectric power station, were selected for the study. One site was located in the main channel of the Jacuí River, and the others in tributaries of its left bank (Figure 1). These are shown in Table 1 (stream order follows Strahler's 1957 classification).

The sampling was conducted in August and November 2001 and in February and May 2002, representing the four seasons of the year (winter, spring, summer, and autumn, respectively). Collections were carried out using a Surber-type sampler (area = 0.36 m^2^) in shallow water (no deeper than 1 m). At each site, three subsamples were taken, one in midstream and one at each bank, except at Site 1, where all the samples were taken at the left bank because of the width of the river. All subsamples from each site were pooled in a single plastic bottle. The macrophytes that were attached on gravel were scraped and added to the samples. The material was fixed in 80% ethanol.

The picking and mounting of the material on slides for identification were done using a stereomicroscope. For taxonomic identification, the specimens were cleared in 10% potassium hydroxide, prepared on semipermanent slides using Hoyer's medium, and examined with an optical microscope. The specimens were identified to species or genus level or were classified as morphotypes using the taxonomic keys of Trivinho-Strixino and Strixino (1995), Cranston (2000), Epler (2001), and Trivinho-Strixino (2011). Identifications were confirmed by Dr. Susana Strixino (Universidade Federal de São Carlos).

Voucher specimens were deposited in the Coleção de Zoologia of the Departamento de Biologia of the Universidade Federal de Santa Maria, Rio Grande do Sul State, and in the Laboratório de Hidrobiologia of the Universidade Federal de São Carlos, São Paulo State.

### Abiotic data

At each sampling site, data for air and water temperatures (Tar and Tag, alcohol 0–50° C thermometer), dissolved oxygen (mg/L oxymeter), acidity (pH, pH meter), depth (m), and water velocity (m/s, float method) were obtained. Data for the cumulative monthly rainfall (mm) and mean monthly regional temperature (° C) were obtained from the Setor de Fitotecnia, Departamento de Zootenia of the Universidade Federal de Santa Maria.

### Data analysis

The richness of the taxa at the four sampling sites was compared using the rarefaction technique (1,000 permutations) (Simberloff 1972). Comparison of richness by means of the rarefaction technique must be done at the lowest level of comparison between communities (Gotelli and Entsminger 2011). Thus, the four sites were compared on the basis of a subsample of 71 randomly drawn specimens. This number corresponds to the smallest number of individuals found at a site. The curves were generated by Ecosim 700 software (Gotelli and Entsminger 2011).

The similarity among the chironomid larvae assemblages from the four sampling sites was evaluated using the Bray-Curtis similarity coefficient with the non-metric multidimensional scaling (NMDS) ordination method (Kruskal and Wish 1978). The stress statistic was used as a measure of the similarity matrix representation by the NMDS ordination. Stress values below 0.2 correspond to a reasonable fit of an ordination (Clarke and Warwick 2001). The ordination of the samples was done in two sets: i) Spatial NMDS: the samples were plotted according to the sampling site; ii) Temporal NMDS: the samples were plotted according to the season of the collection. The analyses were performed using Primer E software (Clarke and Gorley 2006).

The abundance of larvae over time does not increase linearly but rather is a periodic process (Pinheiro et al. 2002). Therefore, the occurrence of a seasonal pattern in the temporal distribution of the abundance and richness of chironomid larvae assemblages was verified by statistical circular analysis (Zar 1999). In this analysis, the four months (seasons) of sampling were transformed into angles of 90° intervals (August 2001 = 0°; November 2001 = 90°; February 2002 = 180°; May 2002 = 270°). Thus, the abundance and richness of chironomid larvae at each site in each season was transformed into the frequency of the corresponding angle (see Prado et al. 2005; Both et al. 2008). For each site, the following parameters were estimated: i) mean vector angle (μ), which represents the time of the year during which the greatest abundance and richness were recorded; ii) circular standard deviation; iii) length of the vector (r), a measure of the concentration of the data along the cycle analyzed (year), of which the value varies from 0 (maximum dispersion of data) to 1 (maximum concentration of data). The significance of the mean angle was determined using Rayleigh's Test (Z) (Zar 1999). The circular analysis was performed using Oriana 3.21 software (Kovach 2010).

The influence of the environmental variables on the spatial and temporal distributions of the chironomid larvae assemblages was analyzed by canonical correspondence analysis (CCA) (Legendre and Legendre 1998) using the software CANOCO (Ter Braak and Šmilauer 2002). This analysis was selected due to the intermediate gradient, i.e., standard deviation length between 3 and 4 (SD = 3.172) shown by the data for composition of the chironomid larvae assemblages (high beta diversity) (*sensu* Ter Braak and Šmilauer 2002).

In the CCA, the following environmental variables were tested through the manual forward stepwise selection procedure (*p* < 0.05 according to the Monte Carlo permutation test with 999 randomizations): pH, dissolved oxygen, water temperature, mean air temperature, depth, water velocity, altitude, and rainfall. Only three of these environmental variables (mean air temperature, altitude, and rainfall) were included in the analysis. This method was also efficient in removing the multicolinearity among the explanatory variables because none of the three selected variables showed a high variance inflation factor (*sensu* Ter Braak and Šmilauer 2002). Rare taxa were down-weighted, and the Monte Carlo test (999 randomizations) was used to test the significance of the canonical axes (Ter Braak and Šmilauer 2002). The biotic data were square-root transformed, and the environmental data were square-root transformed and standardized (by the standard deviation). The data were transformed using an algorithm to normalize them and to make them homoscedastic (Sokal and Rohlf 1995). The environmental data were standardized to homogenize the scale of the different units of measure included in the environmental matrices (e.g., °C for air temperature and mm for rainfall) (Clarke and Gorley 2006).

## Results

The mean values of pH, dissolved oxygen, and air and water temperatures were very similar among the four sampling sites. The pH was slightly acid, and the water was well-oxygenated (Table 2). The depth was slightly greater at Site 4, as was the water velocity at Site 2 (Table 2).

During the different months, the mean pH value varied over a narrow range, and the dissolved oxygen was slightly lower in May (autumn) (Table 2). The air and water temperatures on the sampling days and mean monthly air temperature were higher in November (spring) and February (summer), while the mean depth and mean water velocity were higher in February and May, when the highest values for cumulative rainfall were also recorded (Table 2).

### Spatial structure

In total, 1,816 specimens belonging to 69 taxa were collected (Table 3). *Rheotanytarsus* sp. 1 (24.1%), *Cricotopus* sp. 2 (14.9%), *Rheotanytarsus* sp. 2 (9.1%) and *Polypedilum* (*Polypedilum*) sp. 2 (8.2%) were the most abundant taxa, representing 56.4% of the total. Twenty taxa (approximately 30%) were rare, each represented by fewer than 3 specimens (Table 3).

The abundance and richness varied among the four sampling sites. Site 3 showed the highest abundance and richness (731 larvae and 37 taxa, respectively), while Site 2 showed the lowest abundance (71 larvae). The lowest richness was recorded at Sites 2 and 4 (25 taxa) (Table 3). The dominance also varied among the sites. Over 50% of the specimens from Site 1 were represented by *Cricotopus* sp. 2 (26.6%), *Thienemanniella* sp. 2 (19.6%), and *Cricotopus* sp. 1 (13.7%). At Site 3, the dominant taxa were *Rheotanytarsus* sp. 1 (42.2%) and *Rheotanytarsus* sp. 2 (16.5%), while *Polypedilum* (*Polypedilum*) sp. 2 (21.04%), *Polypedilum* (*Polypedilum*) sp. 1 (17.2%), and *Cricotopus* sp. 2 (14.5%) were dominant at Site 4. At Site 2, no taxa were dominant (Table 3), but higher abundances were shown by *Rheotanytarsus* sp. 1 (16.9%), *Rheotanytarsus* sp. 2 (12.7%), *Polypedilum* (*Polypedilum*) sp. 1 (12.6%), and *Polypedilum* (*Polypedilum*) sp. 2 (11.2%). Five taxa occurred at all four sites (*Polypedilum* (*Polypedilum*) sp. 1, *Rheotanytarsus* sp. 1, *Rheotanytarsus* sp. 2, *Thienemanniella* sp. 1, and *Lopescladius*), while 12 taxa were exclusive to Site 1, 5 to Site 2, 14 to Site 3, and 7 to Site 4 (Table 3).

**Figure 2.**
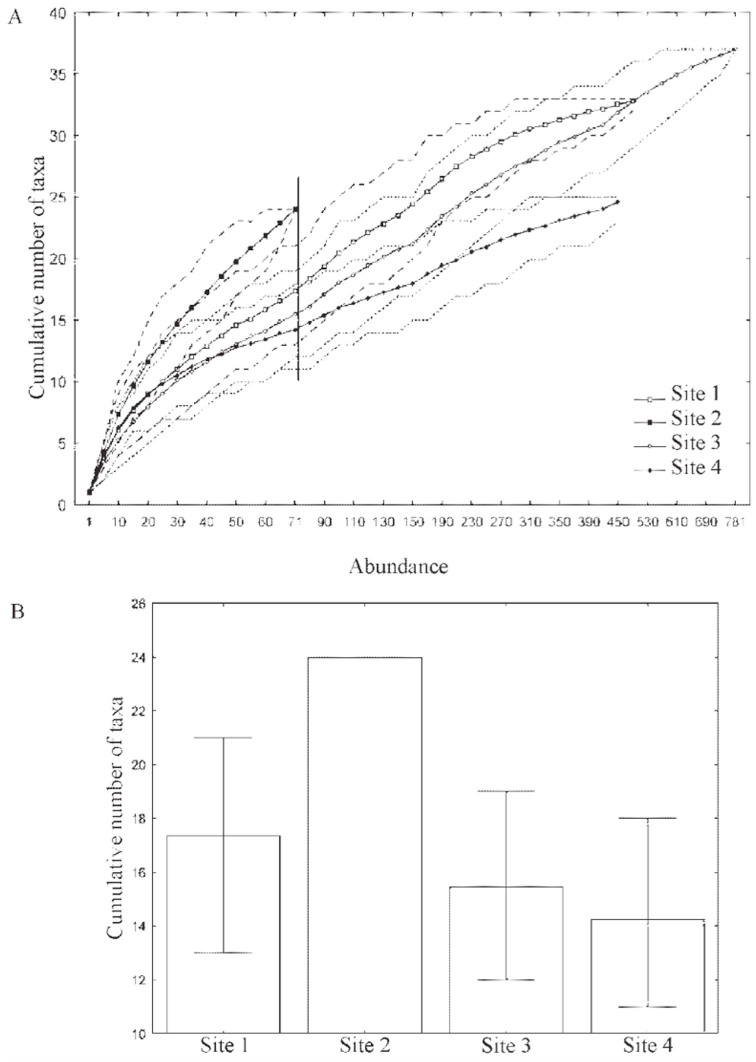
Comparison of the estimated richness of the Chironomidae larvae assemblages among the sampled sites in the middle course of the Jacuí River Basin, RS, Brazil, in the period of August to November 2001 and February to May 2002. A) the rarefaction curves of the estimated richness: the vertical bar represents the comparison point among the four sites, the dotted curves indicate the variation around the average curve, which is in turn represented by the continuous curve; B) point of comparison for a subsample of 71 randomly drawn specimens. The error bars indicate the variation around the average. High quality figures are available online.

The rarefaction technique indicated that Site 2 showed greater richness than the other sites (Figure 2). Sites 1, 3, and 4 did not show any difference in richness and also showed a wide overlap in the variation around the mean of these three sites (Figure 2B). However, if only the curves of the three sites with the greatest chironomid abundance are considered, a new comparison point is assumed (for a sample of 450 randomly drawn specimens), and more information can be obtained. In this scenario, Sites 1 and 3 continued to show wide overlap in the mean curves and in the confidence intervals, and both showed higher richness than Site 4, for which the mean curve was below the others, and the confidence intervals did not overlap at the comparison point (Figure 2A).

**Figure 3.**
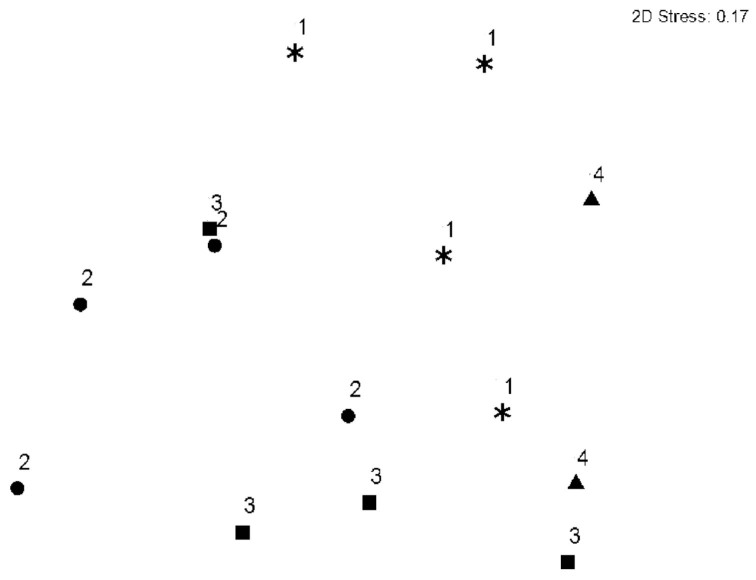
NMDS Ordination diagram of the samples of the Chironomidae larvae assemblages collected at Sites 1, 2, 3, and 4, in the middle course of the Jacuí River Basin. High quality figures are available online.

The NMDS ordination of the samples of the chironomidae larvae assemblages indicated a slight tendency for spatial segregation among the sites (Figure 3). The samples from Sites 2 and 3 showed greater overlap than the samples from Sites 1 and 4, which showed a tendency to form individual groups (Figure 3). Two samples from Site 4 were too distant from the others because of the low abundance and richness of their larvae assemblages and therefore are not shown in the graph.

### Temporal structure

**Figure 4.**
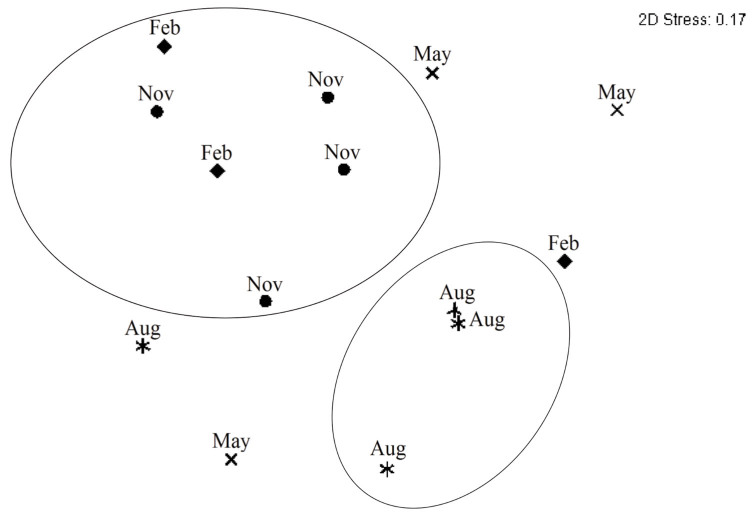
NMDS Ordination Diagram of the seasons of the year surveyed between August (Aug, winter) and November (Nov, spring) 2001 and February (Feb, summer) and May (Autumnl) 2002 in the middle course of the Jacuí River Basin. High quality figures are available online.

The NMDS ordination of the samples of the chironomid larvae assemblages showed a temporal structure within the samples, which tended to form two groups (Figure 4). One group was formed by most of the samples collected during the spring and summer, and the other by the winter samples, while the autumn samples were distributed between the two groups (Figure 4).

**Figure 5.**
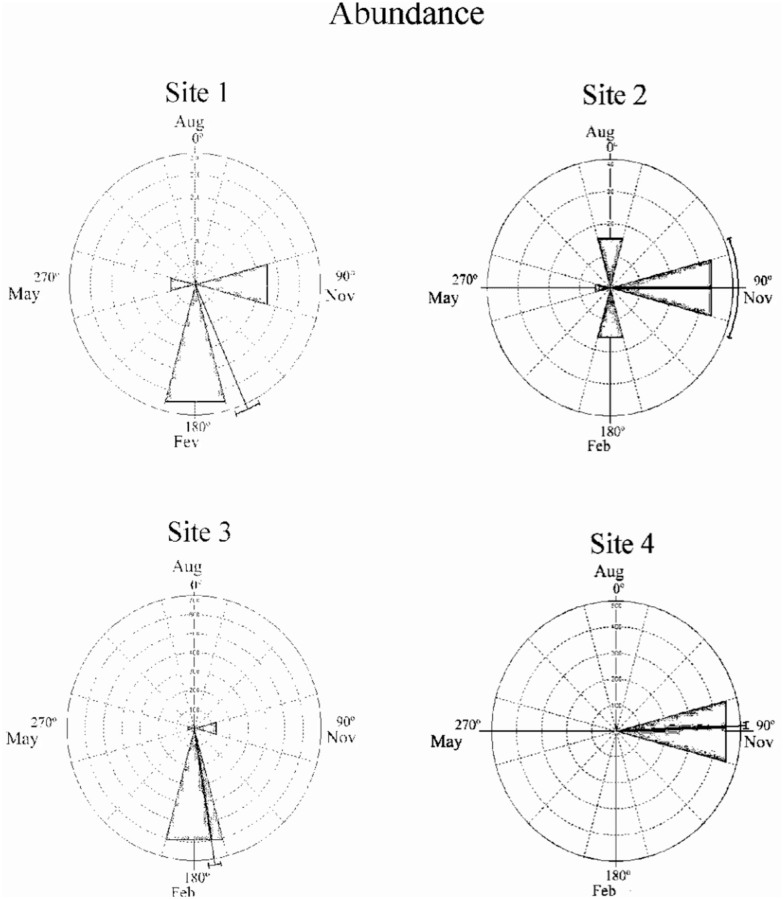
Temporal distribution of the abundance of the Chironomidae larvae assemblages in the middle course of the Jacuí River Basin and its tributaries (Sites 1, 2, 3, and 4), between August (Aug, winter) and November (Nov, spring) 2001 and February (Feb, summer) and May (Autumn) 2002. High quality figures are available online.

**Figure 6.**
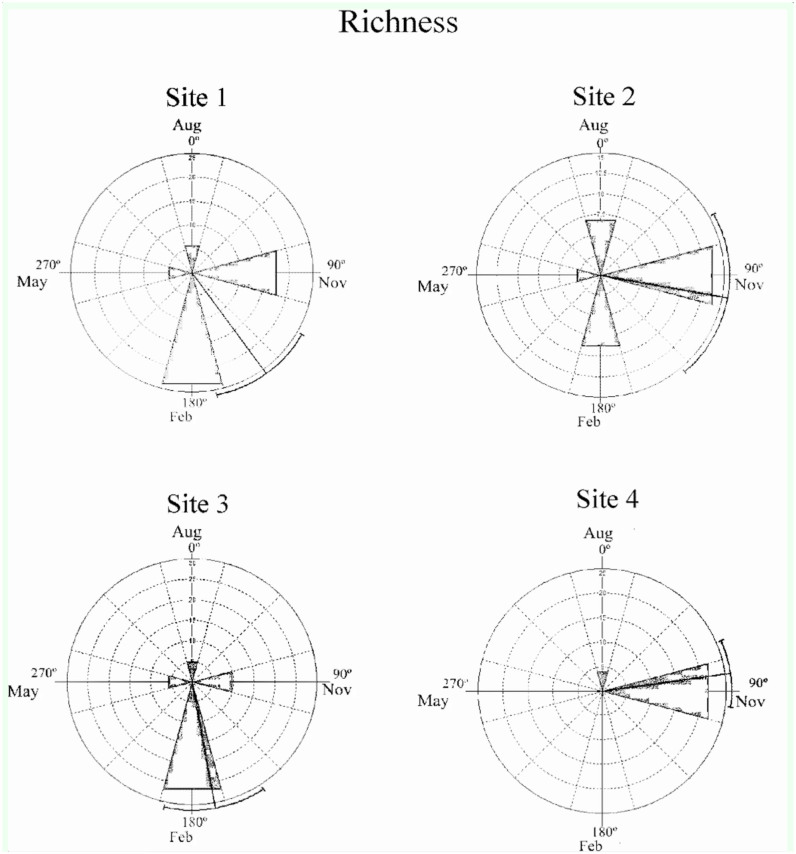
Temporal distribution of the richness of the Chironomidae assemblages in the middle course of the Jacuí River Basin and its tributaries (Sites 1, 2, 3 and 4) between August (Aug, winter) and November (Nov, spring) 2001 and February (Feb, summer) and May (Autumn) 2002. High quality figures are available online.

The temporal structure of the chironomid larvae assemblages detected by NMDS ordination was confirmed by circular analysis. This analysis revealed strong seasonality (r) in the abundance and richness data (Table 4). Rayleigh's test showed statistical significance for the abundance and richness data, which were more prominent during the spring and summer, as shown by the angle of the mean vector (μ) (Figures 5 and 6).

The dominant taxa in each month varied. In August, *Cricotopus* sp. 2 (29.6%), *Rheotanytarsus* sp. 1 (22%), *Polypedilum (Polypedilum*) sp. 1 (11%), and *Thienemanniella* sp. 2 (7.4%) were abundant. In November, *Polypedilum (Polypedilum*) sp. 2 (15%), *Polypedilum (Polypedilum*) sp.l (13%), *Thienemanniella* sp. 2 (13%), *Rheotanytarsus* sp. 1 (12%), *Cricotopus* sp. 2 (9%), and *Rheotanytarsus* sp. 2 (8.3%) were the dominant taxa. In February, *Rheotanytarsus* sp. 1 (33%), *Cricotopus* sp. 2 (18.3%), *Rheotanytarsus* sp. 2 (11%), and *Dicrotendipes* sp. 3 (8%) were the most abundant, while in August, *Cricotopus* sp. (33%), *Rheotanytarsus* sp. 1 (26.4%), and *Cricotopus* sp. 1 (21%) were dominant.

### Influence of the environmental variables on the spatial and temporal structure of the chironomid larvae assemblages

**Figure 7.**
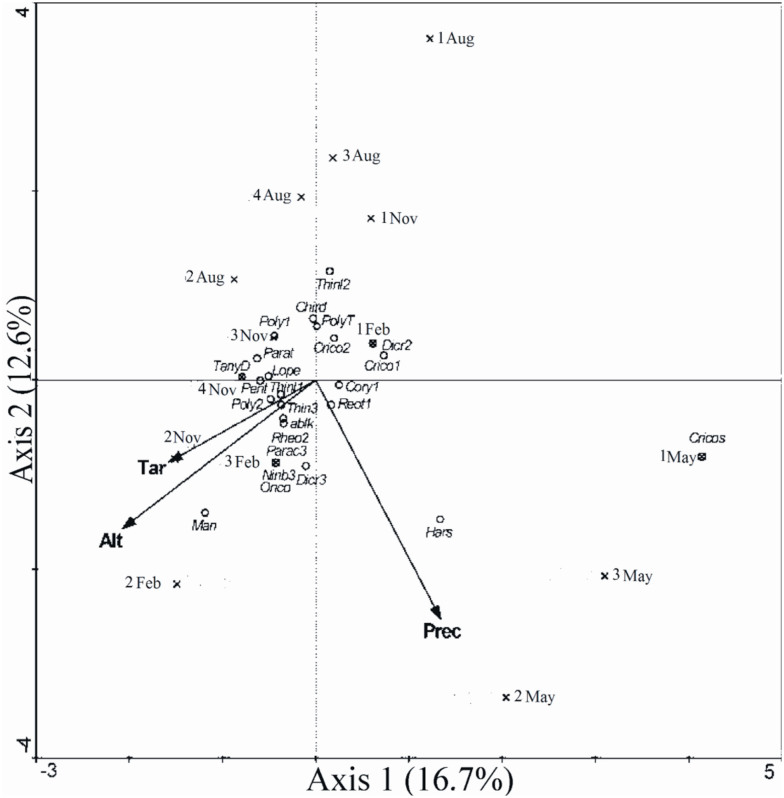
Diagram of ordination of the samples and taxa for the first two axes of the canonical correspondence analysis of Chironomidae larvae assemblages in the middle course of the Jacuí River Basin and environmental variables, surveyed in the months of August (Aug) and November (Nov) 2001, and February (Feb) and May 2002 in the sampling sites (1, 2, 3, and 4). Abbreviations of the taxa: Ablk = *Ablabesmyia (Karelia*), Chird = *Chironomus decorus,* Cory I = *Corynoneura* sp. 1, Cricol = *Cricotopus* sp. 1, Crico2 = *Cricotopus* sp. 2, Cricos = *Cricotopus,* Dicro2 = *Dicrotendipes* sp. 2, Dicro3 = *Dicrotendipes* sp. 3, Hars = *Harnischia* (?) sp.l, Lope = *Lopescladius,* Man = *Manoa,* Nimb3 = *Nimbocera* sp.3, Onco = *Onconeura* sp., Parac3 = *Parachironomus* sp. 3, Parat = *Paratendipes,* Poly 1 = *Polypedilum (Polypedilum*) sp. 1, Poly2 = *Polypedilum (Polypedilum*) sp. 2, PolyT = *Polypedilum (Tripodura*), Rheo2 = *Rheotanytarsus* sp. 2, Rhetl = *Rheotanytarsus* sp. 1, TanyD = *Tanytarsini Gênero* D, Thienll = *Thienemanniella* sp. 1, Thinl2 = *Thienemanniella* sp. 2, Thinl3 = *Thienemanniella* sp. 3. High quality figures are available online.

All the axes of the CCA performed with spatial and temporal data of the assemblages were significantly different from those expected by chance (F = 1.86, *p* < 0.01). The first two CCA axes together represented 29.3% of the variability in the data. Of this, 81.9% was explained by the relationship to the environmental variables (Table 5). The first CCA axis indicated a negative correlation with the monthly air temperature and the altitude and a positive correlation with rainfall (Table 6, Figure 7). The second axis showed a negative correlation with altitude and rainfall and a weak negative correlation with the monthly air temperature (Table 6, Figure 7).

In general, the first CCA axis summarized the spatial structure, while the second axis represented the temporal structure present in the chironomid larvae assemblages of the middle Jacuí River basin. On axis 1, the samples were distributed in a gradient according to the three environmental variables included in the analysis; altitude had the greatest influence. Thus, the samples from Site 2 (at the highest altitude) tended to cluster at one end of the gradient, while the samples from Site 1 (lowest altitude) clustered at the other end (Figure 7). On axis 2, the winter and autumn samples segregated, one at each end of the gradient (Figure 7). This distribution was mainly related to the accumulated precipitation recorded in the months of the collections. The precipitation was low in the winter (August) samples and was highest in the autumn (May) (Table 2).

Several taxa of Chironomidae were influenced by environmental variables (Figure 7). *Harnischia* (?) sp. 1 showed a closer relationship to high precipitation, while *Polypedilum (Polypedilum*) sp. 1, *Paratendipes, Chironomus decorus, Polypedilum (Tripodura*), and *Cricotopus* sp. 2 were positively influenced by low precipitation. *Manoa, Nimbocera,* and *Onconeura* showed a closer relationship to high altitude, and *Cricotopus* sp. 1, *Cricotopus* sp. 2, *Dicrotendipes* sp. 2, and *Thienemanniella* sp. 2 to low altitude.

## Discussion

The slight variations in the abiotic factors analyzed at the sites were probably due to the environmental similarities between the sites. The dissolved oxygen levels were relatively high due to the location in mountain areas. The pH of the Jacuí River is slightly acidic (Siegloch et al. 2008; FEPAM 2010). The slightly higher depth of Site 4 (Carijinho River) was due to the morphology of the channel, which cuts a deep, narrow valley. Site 2, in Lajeado do Gringo, showed the highest water velocity, possibly because of the steeper slope, as this site is located at the highest altitude.

Variations in the abiotic factors were also determined by the temporal scale, i.e., factors related to the monthly mean air temperature and monthly rainfall. Thus, the highest values of rainfall recorded in February and May 2002 correlated with the highest values of depth and velocity measured during these months. Conversely, the low value of rainfall recorded in August 2002 led to the lower water velocity in this month. The higher monthly mean air temperatures recorded in November 2001 and February 2002 correlated with the highest values of air and water temperatures at the sampling sites. On the other hand, the low monthly mean temperature recorded in August 2001 may have led to the high value of dissolved oxygen during this month. An inverse relationship between temperature and dissolved oxygen is recorded in the literature (Ali et al. 2002). However, the low value of dissolved oxygen recorded in May 2002, which also showed a low monthly mean temperature, may have been compensated by the higher rainfall, a relationship discussed in other studies (Pinder 1986; Agostinho et al. 2009).

The richness (69) recorded in the middle Jacuí River basin is one of the highest recorded in Brazil. In fact, an inventory conducted by the senior author in this watershed (Floss et al. 2012), using a wide temporal and spatial scale of sampling, recorded 99 taxa. Similar richness was found in Brazil, only in studies conducted over wider temporal (71 taxa in a 12-month study; Siqueira et al. 2008) and/or spatial (51 taxa in nine small rivers; Corbi and Trivinho-Strixino 2008) scales. The high overall richness in this region agrees with the tendency of riverine chironomid larvae assemblages to show higher richness in temperate regions than in tropical regions (McKie et al. 2005; Raunio 2008; Floss et al. 2012). However, the relatively high richness recorded in this study may be related to the slope-plain transition of the region, as well as to the gravelly substrate of the streams, as discussed by Floss et al. (2012). Transition zones some-times show greater richness because species of mountainous and high-altitude areas can be found together with species of potamic areas (Principe et al. 2008). Many of these species are rare because they live close to their ecological limits (Statzner and Higler 1986). In the study area, the rarity of many taxa was notable and corroborates this assumption. Gravelly substrates also contribute to high richness because they promote habitat heterogeneity, favoring the occurrence of diverse macroinvertebrate (Cogerino et al. 1995; Beisel et al. 2000; Voelz and Mcarthur 2000; Principe and Corigliano 2006) and chironomid (Lindergaard 1995a; Lencioni and Rossaro 2005) faunas.

The dominant taxa in the area (*Rheotanytarsus* sp. 1, *Cricotopus* sp. 2, *Rheotanytarsus* sp. 2, *Polypedilum (Polypedilum*) sp. 2), and/or those that occurred at all sampling sites (*Polypedilum (Polypedilum*) sp. 1, *Thienemanniella* sp. 1, and *Lopescladius*) are represented by genera characteristic of lotic environments with a gravel bottom, litter and fine sediment deposition, and riparian and aquatic vegetation (Sanseverino and Nessimian 2001; Rosa et al. 2011). These characteristics were observed at all the sites, favoring the high abundance of these taxa.

The differences in richness and in the dominant taxa at the sampling sites may be related to environmental features and human activities. Site 1 differs from the others because the stream is 7^th^ order, and its water level is regulated by the Dona Francisca Hydroelectric Power Station, hindering permanent contact between the riverbed and the non-leafy riparian vegetation. The irregular contact of the riparian vegetation with the water, and the consequential small amount of shade, decrease litter input and deposition, as well as other residues on the riverbed, but increase the biomass of periphyton due to greater exposure to light (Jacobsen et al. 2003). Many taxa exclusive to Site 1, such as *Dicrotendipes* sp. 2, *Goeldichironomus pictus*, and *Parachironomus* sp. 2, are typical of lentic waters in the process of eutrophication (Spies et al. 2009), higher-order rivers, potamic areas, or areas with sandy bottoms (Principe et al. 2008). *Cricotopus*, one of the dominant larval genera at Site 1, is found in all freshwater bodies and is a scraper commonly associated with epiphytic algae (Cranston et al. 1983; Berg 1995; Epler 2001).

The other sites are very similar in their environmental characteristics, but Site 4 can be considered the most well-preserved because of its location in a deep valley far from farmhouses. The dominance of *Polypedilum (Polypedilum*) and *Cricotopus* at this site may be related to the site's environmentally well-preserved condition and its greater depth and lower water velocity. The larvae of *Polypedilum* occur in nearly every kind of lentic and lotic environment (Pinder and Reiss 1983) but are preferably associated with organic detritus in deposition areas (backwaters) and/or deeper areas (pools; Sanseverino and Nessimian 1998). Some species of this genus can be found associated with hard substrates and plants (Pinder and Reiss 1983; Sanseverino et al. 1998; Spies et al. 2009). Sites 2 and 3 are environmentally very similar. Both are located near small farmhouses and are affected by human sewage and cattle; however, at Site 2 there is sediment deposited from erosion at one of its banks, where the soil is disturbed by planted fields and riparian vegetation is absent. The absence of *Cricotopus* and *Thienemanniella* from this site may reflect this anthropogenic effect as well as the lack of aquatic vegetation and faster water velocity because both genera are sensitive to these conditions (Galdean et al. 2000; Inoue et al. 2005; Silva et al. 2008). On the other hand, the dominance of *Rheotanytarsus* at Sites 2 and 3 may be related to the environmental similarity of these areas. The larvae of this genus prefer lotic environments (Spies et al. 2009; Rosa et al. 2011) and show a positive relationship to the canopy cover of the riparian vegetation and its shade (Inoue et al. 2005).

The highest richness and abundance of Chironomidae were recorded at Site 3. This site was not as impacted as Site 1, which had a regulated course, but was not as well-preserved as Site 4, due to its proximity to farmhouses. Thus, its greater richness may have been determined by the dense stands of the macrophyte *Podostemun* and also by its intermediate degree of environmental impact. Aquatic vegetation favors a higher diversity of macroinvertebrates because it increases the heterogeneity of the environment (Townsend and Scarsbrook 1997; Taniguchi and Tokeshi 2004). Macrophytes can provide shelter for larvae and foster their development and feeding (Ali et al. 2002; Woodcock et al. 2005). The positive relationship with aquatic vegetation has also been observed for the Chironomidae else-where (Cranston and McKie 2006). In addition, the intermediate degree of impact at Site 3 may have favored the occurrence of a larger number of species due to the coexistence of tolerant and sensitive species (Connell 1978).

The results obtained through the rarefaction technique suggest that the use of a sample of 71 randomly drawn larvae for the comparison among the four sampling sites may not be enough to characterize the larval assemblages present at Sites 1, 3, and 4. These sites showed higher abundances than Site 2. In fact, the rarefaction technique assumes that the communities being compared show the same pattern of abundance and distribution (Gotelli and Colwell 2001). When Site 2 is eliminated from the comparison and a randomly-drawn 450-specimen sample is adopted, it is possible to find differences among the richness of the three sites. The higher standard richness recorded for Sites 1 and 3 can be explained by the intermediate degree of environmental impact and macrophytes at Site 3 and because Site 1 is in an area of transition between mountainous and potamic zones, as discussed above. Site 4 can be considered the most well-preserved site because it is located in a deep and narrow valley where there was less human interference.

The spatial segregation among the groups formed by Sites 2 and 3 and Sites 1 and 4 can be related to differences in some of their landscape features. The former group is represented by its location in middle-order (3^rd^ and 4^th^) stretches. Middle-order rivers, according to the river continuum concept (Vannote et al. 1980), show greater richness and functionally shared fauna. Some studies on the longitudinal gradient of rivers, focusing on different orders, have shown that chironomid larvae assemblages are richer in middle-order stretches (Lindegaard 1995b; Principe et al. 2008; Puntí et al. 2009; Chaib et al. 2011). Sites 2 and 3 are also subject to moderate disturbance, which would likely result in a richer fauna (Townsend 1989). The latter group (Sites 1 and 4) consists of sites with marked environmental differences. While Site 4 was the most well-preserved, Site 1 was the most impacted, as it had a regulated flow and is located in a 7^th^-order stream at the lowest altitude. Assemblages of Chironomidae in large rivers, close to potamic areas and/or with flow affected by dams, show particular taxonomic compositions (Principe et al. 2008; Rosin et al. 2009).

The closer distribution of winter (August) samples in the NMDS ordination indicated that the season might be a critical factor influencing the temporal distribution of the chironomid larvae assemblages. The concentration of abundance and richness in the spring (November) and summer (February) confirms this. Seasonal changes in abundance and richness of chironomid larvae have been reported previously. In the Northern Hemisphere, higher abundance and richness of larvae and adults have been found in the spring and summer (Ali 1980; Spänhoff et al. 2004; Reynolds and Benke 2005; Steven et al. 2005; Boulton et al. 2008). In several regions of the world, the seasons are also characterized by differences in precipitation. Most tropical regions in Brazil, such as the Amazon, Caatinga, Cerrado, and Pantanal biomes, undergo periods of intense rain during summer, (Morrone 2006). However, in some regions, such as in the study area, periods of high precipitation or intense drought can occur in any month of the year (Maluf 2000). In our study, both the lowest temperature and the lowest rainfall were recorded during the winter (August). Autumn (May) was as cold as winter but had the highest rainfall. This difference in precipitation may have generated the differences in the samples of the winter and autumn as evidenced by NMDS ordination. Thus, although the variations in temperature and precipitation throughout the year were not very intense or regular, as in many temperate regions of the Northern Hemisphere, they seemed to be sufficient to influence the structure of the chironomid larvae assemblages in southernmost Brazil. On the other hand, temperature and precipitation also influenced many abiotic factors related to variations in the spatial distribution of the assemblages studied.

The influence of abiotic factors, such as air temperature, rainfall, and especially altitude, on the spatial and temporal structure of the chironomid larvae assemblages was confirmed by the CCA. There is no information in the literature regarding the altitude preferences of *Manoa, Nimbocera,* or *Onconeura.* However, species of the genera *Cricotopus, Dicrotendipes,* and *Thienemanniella* have been found in lowland rivers at low altitudes (Principe et al. 2008; Puntí et al. 2009; Chaib et al. 2011), as was observed in our study. *Harnischia* is tolerant to variations in some environmental factors and can occur in deep and turbulent waters associated with sandy sediment (Epler 2001; Resende and Takeda 2007), which indirectly agrees with its relationship to high rainfall levels. *Polypedilum (Polypedilum), Paratendipes, Chironomus decorus, Polypedilum (Tripodura*), and *Cricotopus* are associated with shallow and calmer waters that are rich in organic matter and aquatic vegetation and have coarse sand and periods of low water levels (Takeda et al. 1997; Sanseverino and Nessimian 1998) in agreement with their preference for low precipitation.

## Conclusions

Studies conducted with freshwater macroin-vertebrates have shown that the spatial scale adopted to analyze communities affects the results regarding the influence of certain environmental factors on their distribution (Principe et al. 2008). Even though the present study has focused on small-scale analyses, factors traditionally associated with landscape features (such as altitude, preservation of riparian vegetation, and stream order) may influence the distribution on a local scale. However, as only one site in a high-order stream was sampled, and this site was affected by a dam, it is difficult to precisely evaluate the importance of this factor on a small spatial scale in the study area.

Factors such as temperature and rainfall, as well as related variables (e.g., depth and water velocity), may interact and influence the assemblages spatially and temporally. In addition, the influence of factors such as altitude, temperature, and rainfall on specific taxa confirms that many species of Chironomidae and their assemblages are sensitive to environmental conditions. The present study also demonstrates that both the environmental preservation of the riparian vegetation and the habitat heterogeneity (coarse granulometry and aquatic macrophytes) are important for the conservation of the chironomid larvae assemblages and, consequently, for the maintenance of the integrity of riverine biota. However, the environmental conditions that favor the diversity of Chironomidae, which are one of the most important groups for the maintenance of the food chains of limnetic ecosystems, are being affected by dam construction, which alters the river bottom and drowns the riparian vegetation. Although the samples used in this study were collected about a decade ago, the environmental conditions in the middle Jacuí River basin remain similar in the present day. The area around the Dona Francisca Hydroelectric Power Station and its reservoir was incorporated into a state park (Parque Estadual da Quarta Colônia), which guarantees the ecological integrity of a considerable portion of the region. Thus, additional taxonomic and ecological studies are necessary in order to allow the use of chironomid larval assemblages in environmental monitoring programs in the region.

**Table 1.**
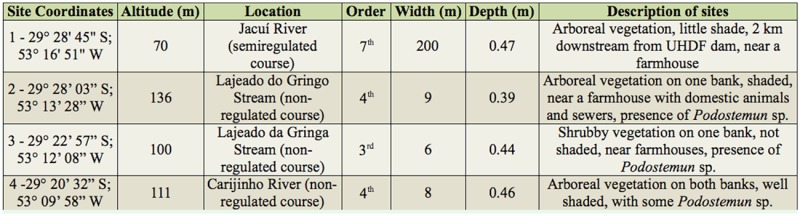
Location and characterization of the sampling sites of the Chironomidae larvae assemblages sampled between April 2000 and May 2002 in the middle course of the Jacuí River, RS, Brazil.

**Table 2.**
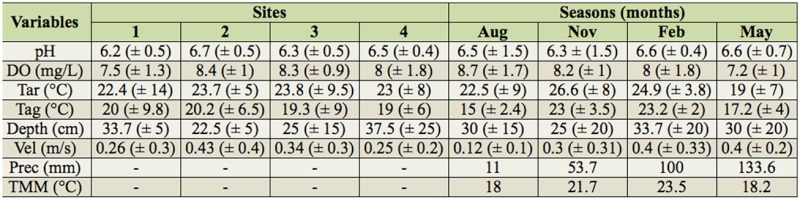
Average values and standard deviation of environmental variables (pH, DO = dissolved oxygen, Tar = air temperature, Tag = water temperature, Depth, Vel = water velocity, Prec = precipitation, TMM = mean monthly air temperature) at the four sampling sites (1/Jacuí River, 2/Lajeado do Gringo; 3/Lajeado da Gringa; 4/Carijinho River) and during four seasons (winter, Aug/01; spring, Nov/01; summer, Feb/02; autumn, May/02), measured in the middle course of the Jacuí River Basin, RS, Brazil.

**Table 3.**
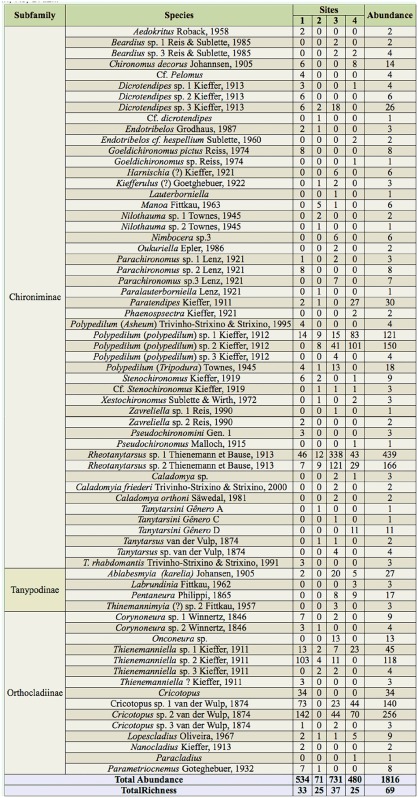
Taxonomic composition and abundance of Chironomidae larvae found at the four sampling sites in the middle course of the Jacuí River Basin, RS, Brazil.

**Table 4.**
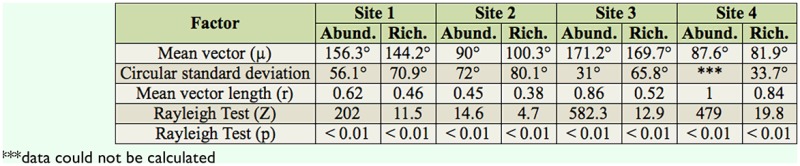
Circular analysis of the abundance (Abund.) and richness (Rich.) of the Chironomidae larvae assemblages in the middle course of the Jacuí River Basin, sampled in August and November 2002 and February and May 2002.

**Table 5.**
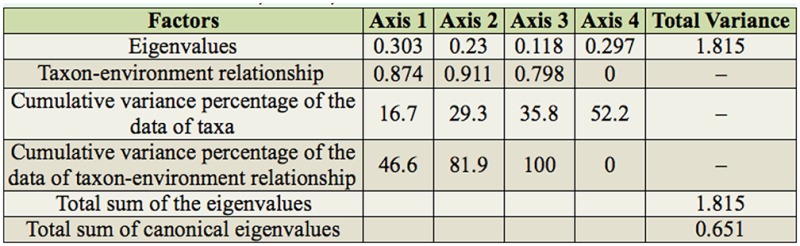
Eigenvalues, taxon-environment coefficients of correlation, and explained cumulative percentage of the four first axes of the canonical correspondence analysis of the Chironomidae larvae assemblages of the middle course of the Jacuí River Basin, sampled in August and November 2011 and February and May 2002.

**Table 6.**
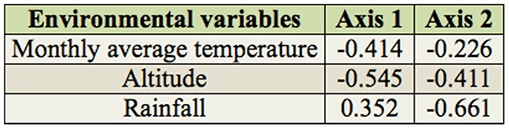
Inter-set correlations between the first two axes of the canonical correspondence analysis and the environmental variables of the Chironomidae larvae assemblages in the middle course of the Jacuí River Basin, sampled in August and November 2011 and February and May 2002.

## Abbreviations

CCA,: canonical correspondence analysis;
NMDS,: non-metric multidimensional scaling
